# Rho kinase and Na^+^/H^+^ exchanger mediate endothelin‐1‐induced pulmonary arterial smooth muscle cell proliferation and migration

**DOI:** 10.14814/phy2.13698

**Published:** 2018-05-13

**Authors:** John C. Huetsch, Jasmine Walker, Clark Undem, Julie Lade, Xin Yun, Syeda Baksh, Haiyang Jiang, Ning Lai, Larissa A. Shimoda

**Affiliations:** ^1^ Division of Pulmonary and Critical Care Medicine Department of Medicine Johns Hopkins School of Medicine Baltimore MD

**Keywords:** Endothelin, pulmonary arterial smooth muscle, Rho kinase, sodium‐hydrogen exchanger

## Abstract

Excessive production of endothelin‐1 (ET‐1) has been observed in almost all forms of pulmonary hypertension. ET‐1, a highly potent vasoconstrictor, can also potentiate pulmonary arterial smooth muscle cell (PASMC) growth and migration, both of which contribute to the vascular remodeling that occurs during the development of pulmonary hypertension. Increasing evidence indicates that alkalinization of intracellular pH (pH_i_), typically due to activation of Na^+^/H^+^ exchange (NHE), is associated with enhanced PASMC proliferation and migration. We recently demonstrated that application of exogenous ET‐1 increased NHE activity in murine PASMCs via a mechanism requiring Rho kinase (ROCK). However, whether ROCK and/or increased NHE activity mediate ET‐1‐induced migration and proliferation in PASMCs remains unknown. In this study, we used fluorescent microscopy in transiently cultured PASMCs from distal pulmonary arteries of the rat and the pH‐sensitive dye, BCECF‐AM, to measure changes in resting pH_i_ and NHE activity induced by exposure to exogenous ET‐1 (10^−8^ mol/L) for 24 h. Cell migration and proliferation in response to ET‐1 were also measured using Transwell assays and BrdU incorporation, respectively. We found that application of exogenous ET‐1 had no effect on NHE1 expression, but increased pH_i_, NHE activity, migration, and proliferation in rat PASMCs. Pharmacologic inhibition of NHE or ROCK prevented the ET‐1‐induced changes in cell function (proliferation and migration). Our results indicate that ET‐1 modulates PASMC migration and proliferation via changes in pH_i_ homeostasis through a pathway involving ROCK.

## Introduction

Three isoforms of endothelin (ET‐1, ET‐2, ET‐3) have been identified, of which ET‐1 is the most widely distributed (Rubin 2012). Primarily produced in, and secreted from, vascular endothelium, ET‐1 is one of the most potent and abundant endothelial‐derived constricting factors identified to date. In the lung, both the synthesis and release of ET‐1 are upregulated under a variety of conditions. In particular, ET‐1 is an important contributor to the vascular changes that occur in the pulmonary circulation in response to hypoxia, as inhibitors of ET‐1 receptors prevent and in some cases reverse, the elevations in pulmonary arterial pressure and vascular remodeling that occur in animal models of hypoxic pulmonary hypertension (PH) (Eddahibi et al. 1995; Chen et al. 1997). Increased ET‐1 levels are observed in other animal models of PH, including the Fawn‐Hooded rat (Stelzner et al. 1992), monocrotaline‐treated animals (Frasch et al. 1999), and the SU5416‐hypoxia rat (Rafikova et al. 2013), as well as in patients with PH of varied etiologies, including pulmonary arterial hypertension (Giaid et al. 1993), chronic thromboembolic PH (Bauer et al. 2002), congenital heart disease (Ishikawa et al. 1995), and persistent pulmonary hypertension of the newborn (Rosenberg et al. 1993).

Regardless of etiology, increased muscularization of the vasculature, due to both proliferation of existing smooth muscle and extension of muscle along the vascular tree to the precapillary arterioles, contributes to the elevation in pulmonary arterial pressure (Shimoda and Laurie 2013). Several studies have shown that in addition to causing contraction, ET‐1 can also have mitogenic properties (Janakidevi et al. 1992; Zamora et al. 1996; Wort et al. 2001). The exact mechanisms by which ET‐1 promotes pulmonary arterial smooth muscle cell (PASMC) growth are not well understood, but we and others have demonstrated that increased intracellular pH (pH_i_) accompanies cell proliferation in systemic (Bobik et al. 1991; Mitsuka et al. 1993; LaPointe and Batlle 1994) and pulmonary (Quinn et al. 1996; Walker et al. 2016) vascular smooth muscle cells. In PASMCs, pH_i_ homeostasis is maintained in large part by several membrane bound transporters, including the Na^+^/H^+^ exchanger (NHE) (Quinn et al. 1991; Rios et al. 2005). Na^+^/H^+^ exchangers are a family of plasma membrane spanning proteins that use the transmembrane Na^+^ gradient to extrude protons (Huetsch and Shimoda 2015), and NHE isoform 1 (NHE1) is the predominant isoform found in PASMCs (Shimoda et al. 2006). We recently showed that ET‐1 activates NHE activity in murine PASMCs (Undem et al. 2012), adding it to a growing list of stimuli, including acute (Madden et al. 2001) and chronic hypoxia (Rios et al. 2005), and growth factors (Quinn et al. 1996), that induce PASMC alkalinization. In that study, we found that ET‐1‐induced activation of NHE was dependent upon Rho kinase (ROCK) (Undem et al. 2012). Inhibition of Na^+^/H^+^ exchange with amiloride analogs (Quinn et al. 1998) or selective knockdown of NHE isoform 1 (NHE1) (Yu et al. 2008; Walker et al. 2016) attenuates vascular remodeling and pulmonary hypertension in rodents exposed to chronic hypoxia. Pharmacologic inhibition of NHE (Quinn et al. 1996; Huetsch et al. 2016; Walker et al. 2016), as well as NHE1 knockdown (Yu and Hales 2011), mitigates enhanced PASMC proliferation and migration in response to a range of stimuli, suggesting that the effect of NHE on vascular remodeling is mediated by alterations in PASMC function. Taken together, the results from these studies indicate that ET‐1, via ROCK, modulates pH_i_ and NHE activity, and also that NHE activation is necessary for altered PASMC function and vascular remodeling in PH. However, whether ROCK and/or increased NHE activity mediate ET‐1‐induced migration and proliferation in PASMCs remains unknown. Therefore, in this study, we tested the hypothesis that ET‐1 increases PASMC proliferation and migration via a pathway that involves ROCK and NHE.

## Methods

### Isolation of pulmonary arterial smooth muscle cells

All procedures were approved by the Animal Care and Use Committee of The Johns Hopkins University School of Medicine. The method for obtaining primary cultures of rat PASMCs has been described previously (Huetsch et al. 2016). Briefly, male Wistar rats were anesthetized with sodium pentobarbital (130 mg/kg i.p.) and the lungs were removed and placed in HEPES‐buffered salt solution (HBSS) containing (in mmol/L): 130 NaCl, 5 KCl, 1.2 MgCl_2_, 1.5 CaCl_2_, 10 N‐[2‐hydroxyethyl]piperazine‐N’‐[2‐ethanesulfonic acid] (HEPES) and 10 glucose, with pH adjusted to 7.2 with 5 mol/L NaOH. Distal (≥5th generation) intralobar pulmonary arteries (200–600 *μ*m outer diameter) were dissected free from connective tissue in ice cold HBSS. The arteries were opened and the lumen gently rubbed to remove the endothelium. Tissue was allowed to recover for 30 min in cold (4°C) HBSS followed by 20 min in reduced‐Ca^2+^ HBSS (20 *μ*mol/L CaCl_2_) at room temperature. The tissue was digested for 20 min at 37°C in an enzyme solution consisting of reduced‐Ca^2+^ HBSS with collagenase (type I; 1750 U/mL, Worthington, Lakewood, NJ), papain (12.35 U/mL, Sigma, St. Louis, MO), bovine serum albumin (2 mg/mL), and dithiothreitol (1 mmol/L). After digestion, trituration with a wide‐bore transfer pipette was used to disperse single smooth muscle cells in Ca^2+^‐free HBSS. For pH, proliferation, and migration assays, PASMCs were placed in basal media (Ham's F‐12 media supplemented with 0.5% FBS and 1% penicillin/streptomycin) and used within 72 h. For mRNA and protein isolation, PASMCs were expanded in Ham's F‐12 media supplemented with 10% fetal bovine serum (FBS) and 1% penicillin/streptomycin, and were then placed in basal media for 24 h prior to mRNA or protein extraction.

### Immunofluorescence

**Figure 1:**
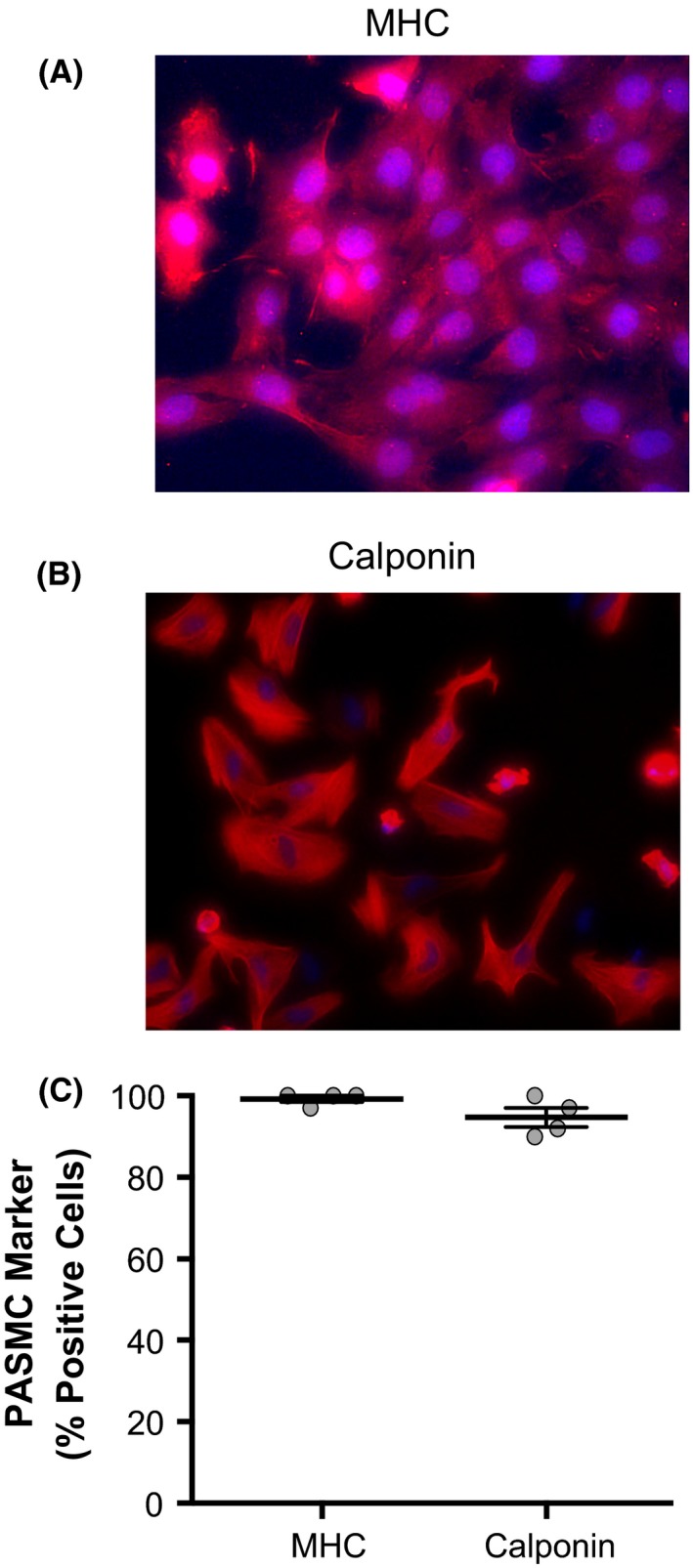
Cells isolated from rats stain positive for pulmonary arterial smooth muscle cell (PASMC) markers. Representative images of cells isolated from rat pulmonary arteries stained for (A) myosin heavy chain (MHC) or (B) calponin, each in red, with DAPI nuclear counterstain in blue. (C) Plot shows percent of DAPI‐positive cells which also stained positive for either MHC or calponin (*n* = 4 for each). Bars show mean with standard error.

PASMCs grown on glass slides were washed, fixed, and permeabilized. Cells were then incubated with antibodies against myosin heavy chain 11 (MHC; Abcam, Cambridge, UK) or calponin (Abcam) followed by incubation with fluorescent‐conjugated secondary antibody (Cy3 goat anti‐mouse IgG; Life Technologies, Carlsbad, CA) plus DAPI nuclear counterstain, and then observed using a microscope with fluorescence objectives (Olympus IX51, Center Valley, PA). The percent of cells which were verified as PASMCs was measured as the ratio of the number of cells positive for PASMC marker to the number of cells positive for DAPI (Fig. 1).

### pH_i_ measurements

PASMCs were placed in a laminar flow cell chamber perfused with either Krebs bicarbonate‐buffered physiologic salt solution (KRBS) containing (in mmol/L): 118 NaCl, 4.7 KCl, 0.57 MgSO_4_, 25 NaHCO_3_, 1.18 KH_2_PO_4_, 2.5 CaCl_2_, and 10 glucose, gassed with 16% O_2_‐5% CO_2_, or with a HEPES‐buffered saline solution (HEPES1) containing (in mmol/L): 130 NaCl, 5 KCl, 1 MgCl_2_, 1.5 CaCl_2_, 10 glucose, and 20 HEPES with pH adjusted to 7.4 with NaOH, as described previously (Huetsch et al. 2016). Cells were incubated with the membrane permeant (acetoxymethyl ester) form of the pH‐sensitive fluorescent dye 2’,7’ ‐bis(carboxyethyl)‐5(6)‐carboxyflourescein (BCECF‐AM, Life Technologies) for 60 min at 37°C under an atmosphere of 21% O_2_‐5% CO_2_. Cells were then washed with KRBS or HEPES1 for 15 min at 37°C to remove extracellular dye and allow complete de‐esterification of cytosolic dye. Ratiometric measurement of BCECF fluorescence was performed on a workstation (Intracellular Imaging, Cincinnati, OH) consisting of a Nikon TSE 100 Eclipse inverted microscope with epifluorescence attachments. Light from a xenon arc lamp was alternately filtered by 490 nm and 440 nm interference filters, and focused onto PASMCs via a 20× fluorescence objective (Super Fluor 20, Nikon, Melville, NY). A filter cube was used to collect light emitted from the cell at 530 nm. Filtered light was then returned through the objective and detected by a cooled CCD imaging camera. Between measurements, an electronic shutter (Sutter Instruments, Novato, CA) was used to minimize photobleaching of dye. All protocols were performed and data collected online with InCyte software (Intracellular Imaging). The ratio of 490 nm to 440 nm emission was calculated by the software and pH_i_ was estimated from in situ calibration after each experiment. For calibration, cells were perfused with a high K^+^ solution containing (in mmol/L): 105 KCl, 1 MgCl_2_, 1.5 CaCl_2_, 10 glucose, 20 HEPES, and 0.01 nigericin to allow pH_i_ to equilibrate to external pH. A two‐point calibration was created from fluorescence measured with pH_i_ adjusted to 6.5 and 7.5.

For resting pH_i_ measurements, PASMCs were treated with 10^−8^ mol/L ET‐1 or vehicle for 24 h, and then were loaded with BCECF and placed on the fluorescence microscope. For bicarbonate‐containing experiments (HCO_3_‐buffered), cells were perfused at a rate of 0.75 mL/min in KRBS solution for >5 min followed by perfusion with calibration solutions. For bicarbonate‐free experiments (HEPES‐buffered), cells were perfused at the same rate in HEPES1 solution for >5 min followed by perfusion with calibration solutions.

To measure NHE activity in bicarbonate‐free conditions (HEPES‐buffered), a standard ammonium pulse technique was used. PASMCs, which had been treated with 10^−8^ mol/L ET‐1 or vehicle for 24 h, were loaded with BCECF, placed on the fluorescence microscope, and perfused at a rate of 0.75 mL/min with HEPES1 solution for 2 min. PASMCs were then briefly exposed to NH_4_Cl (ammonium pulse) by perfusing with HEPES2 solution containing (in mmol/L): 110 NaCl, 20 NH_4_Cl, 5 KCl, 1 MgCl_2_, 1.5 CaCl_2_, 10 glucose, and 20 HEPES at a pH of 7.4 using NaOH for 3 min. The ammonium pulse caused alkalinization due to influx of NH_3_ and buffering of intracellular H^+^. Washout of NH_4_Cl in the absence of extracellular Na^+^ using a Na^+^‐ and NH_4_^+^‐free solution (HEPES3) containing (in mmol/L): 130 choline chloride, 5 KCl, 1 MgCl_2_, 1.5 CaCl_2_, 10 glucose, and 20 HEPES at a pH of 7.4 using KOH for 10 min resulted in acidification due to rapid diffusion and washout of NH_3_, leaving behind H^+^ ions. The external solution was then switched back to HEPES1 solution for 10 min. Re‐addition of extracellular Na^+^ allowed activation of NHE and recovery from acidification to basal levels. NHE activity was measured as the rate of Na^+^‐dependent recovery from intracellular acidification in the first 2 min after re‐exposure to HEPES1. Each experiment concluded with perfusion with calibration solutions.

### Cell migration

Equal numbers of cells (50,000) were seeded onto polycarbonate Transwell inserts (Corning, NY) with transparent, permeable (8 *μ*m pore) filters. Cells were placed in 4 mL of Ham's F‐12 media supplemented with 0.5% FBS and 1% penicillin/streptomycin. Cells were allowed to adhere for 1–2 h before addition of drugs. After 24 h of drug (or vehicle) treatment, filters were washed with PBS, and cells fixed in ice cold 95% ethanol for 10 min. Following fixation, cells were washed with PBS and stained with brilliant blue (Sigma) for 5 min at room temperature. Filters were washed and placed in PBS. Each filter was examined via a microscope‐mounted camera under 4× magnification and images of at least five fields per filter were obtained at random using Q‐capture software to measure total number of cells. The top of the filters were then scraped with a cotton swab, rinsed with PBS to remove nonadherent cells, and images of at least five fields containing migrated cells were again obtained at random. Once images of all fields were obtained, the number of cells in each image was counted. Cell migration was expressed as a percent of total cells.

### Cell proliferation

Cell proliferation was determined using a commercially available ELISA kit for BrdU incorporation (GE Healthcare Life Sciences, Pittsburgh, PA). Each well was plated with 5000 cells in Ham's F‐12 media supplemented with 0.5% FBS and 1% penicillin/streptomycin. Cells were allowed to attach for 1–2 h after cell seeding before inhibitors were added. Thirty minutes after inhibitors were added, BrdU and ET‐1 were then placed in each well. PASMCs were then incubated for 24 h before BrdU incorporation was measured.

### RNA

RNA was extracted from PASMCs using the RNeasy Plus Mini Kit (Qiagen). Reverse transcription was performed using 500 ng of RNA and the iScript cDNA synthesis kit (Bio‐Rad). For comparing expression levels between conditions, quantitative real‐time PCR was performed on resultant cDNA with QuantiTect SYBR Green PCR Master Mix (Qiagen) in an iCycler real‐time PCR detection system (Bio‐Rad). Primer pairs utilized for NHE1 were 5’‐CTCTGATGGAGCTGTGGTGA‐3’ and 5’‐GGGCTGCTACCTGTTCTCAG‐3’. Primer pairs utilized for cyclophilin B were 5’‐GGACGAGTGACCTTTGGACT‐3’ and 5’‐TGACACGATGGAACTTGCTG‐3’. PCR products were confirmed by: 1) a single peak in the melt curve; 2) a single band of the correct size when products were run on an agarose gel; and 3) sequencing of the product. Relative concentrations of each gene were calculated using the Pfaffl method, and data are expressed as a power ratio of the gene of interest to the housekeeping gene (cyclophilin B) within a sample using the efficiency for each gene calculated from efficiency curves run on the same plate as samples. For each experiment, the values were normalized to the value of the vehicle‐treated sample.

### Immunoblot analysis

PASMCs in culture were washed with PBS and total protein was then extracted in ice cold T‐PER buffer (Pierce) containing protease inhibitors (Roche Diagnostics). Proteins were quantified using the BCA protein assay (Pierce) and 10 *μ*g total protein were resolved on SDS‐PAGE gels and transferred to polyvinylidene difluoride membranes. Membranes were blocked in 5% milk and probed with anti‐NHE1 antibody (US Biological, Salem, MA) overnight at 4°C, washed, and incubated in peroxidase‐labeled secondary antibody (KPL) for 1 h. Bands were visualized by enhanced chemiluminescence according to the manufacturer's instructions. Membranes were then probed for *α*‐smooth muscle actin (Sigma), as a loading control. Densitometry was performed in ImageJ to quantify the amount of protein, and the ratio of NHE1 to *α*‐actin was calculated. Fold induction was determined by setting the ratio of NHE1/*α*‐actin in vehicle‐treated cells equal to 1.

### Drugs and chemicals

ET‐1 (EMD Millipore) was used at 10^−8^ mol/L. The vehicle for ET‐1 was diH_2_O. 10 *μ*mol/L Y‐27632 (Cayman Chemical, Ann Arbor, MI) was used to inhibit ROCK. The vehicle for Y‐27632 was diH_2_O. 10 *μ*mol/L ethyl‐isopropyl amiloride (EIPA; Sigma) or 1 *μ*mol/L dimethyl amiloride (DMA; Sigma) was used to inhibit NHE. The vehicle for both EIPA and DMA was dimethyl sulfoxide (DMSO).

### Statistical analysis

Data are expressed as means ± SE. For all data presented, “*n*” represents the number of biological replicates. Since each experiment used cells from a different animal, *“n”* is also the number of animals. For experiments in which pH_i_ was measured, data were collected from 10 to 30 cells per experiment and averaged to obtain a single value for each animal. Statistical comparisons were performed using Student's *t* test or ANOVA, as appropriate. For ANOVA analyses, multiple comparisons testing was performed *post hoc* using the Tukey test. Nonparametric data were transformed prior to analysis. Differences were considered to be significant when *P *<* *0.05.

## Results

### ET‐1 effect on NHE activity and pH_i_ in PASMCs

**Figure 2:**
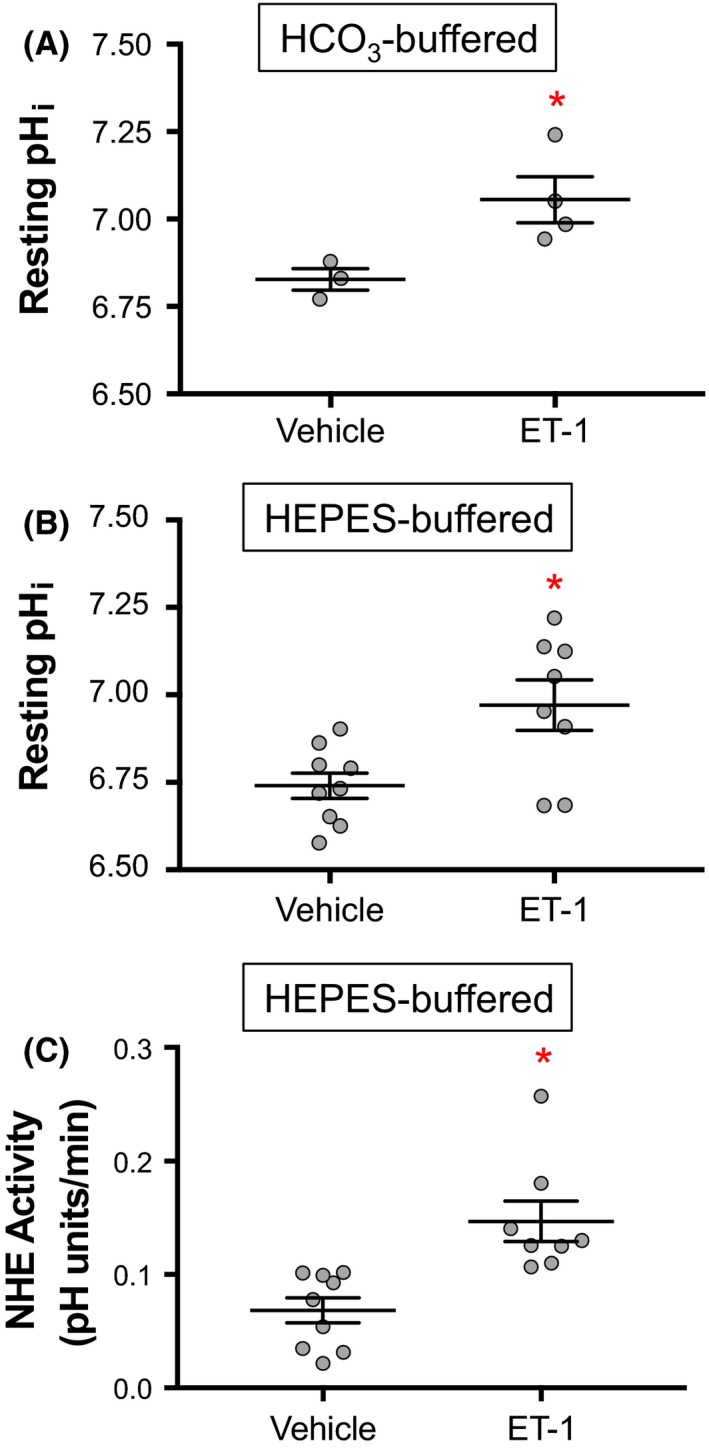
Effect of ET‐1 on resting pH_i_ and Na^+^/H^+^ exchanger (NHE) activity in rat pulmonary arterial smooth muscle cells (PASMCs). Plots show resting pH_i_ of PASMCs treated for 24 h with 10^−8^ mol/L ET‐1 (*n* = 4–8) or vehicle (*n* = 3–9) in (A) bicarbonate‐containing (Krebs) and (B) bicarbonate‐free (HEPES) solutions. (C) Plot shows NHE activity measured via ammonium pulse protocol in PASMCs treated for 24 h with 10^−8^ mol/L ET‐1 (*n* = 8) or vehicle (*n* = 9). Bars show mean with standard error. *Indicates significant difference between groups (*P* < 0.05 by *t* test).

Given that acute exposure (i.e., 15 min) to ET‐1 has been shown to increase resting pH_i_ and NHE activity in PASMCs (Undem et al. 2012), we wanted to determine whether more prolonged ET‐1 exposure resulted in a similar effect. In a physiologic, bicarbonate‐containing solution, resting pH_i_ was 6.83 ± 0.03 units, which was increased to 7.06 ± 0.07 units after 24 h treatment with ET‐1 (Fig. 2A). In order to eliminate the effects of pH_i_‐modulating bicarbonate transporters and isolate the effect of NHE on pH_i_, we measured resting pH_i_ in bicarbonate‐free solution (HEPES). In HEPES, resting pH_i_ was 6.74 ± 0.04 units and was elevated to 6.97 ± 0.07 units after 24 h incubation with ET‐1 (Fig. 2B). Consistent with its effect on pH_i_, ET‐1 also increased NHE activity to 0.15 ± 0.02 pH units/min, compared to 0.07 ± 0.01 pH units/min in vehicle‐treated PASMCs (Fig. 2C). Thus, similar to the acute effects of ET‐1, prolonged exposure to ET‐1 increased resting pH_i_ and NHE activity in PASMCs.

### ET‐1 effect on PASMC function

**Figure 3:**
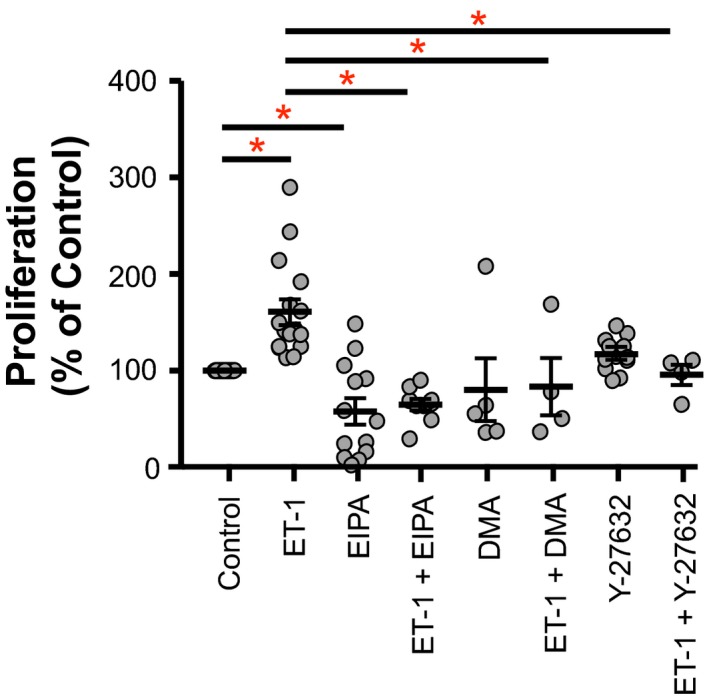
Effect of Na^+^/H^+^ exchanger (NHE) and Rho kinase inhibition on ET‐1‐induced proliferation of rat pulmonary arterial smooth muscle cells (PASMCs). Plot shows effect of 10 *μ*mol/L ethyl‐isopropyl amiloride (EIPA), 1 *μ*mol/L dimethyl amiloride (DMA), or 10 *μ*mol/L Y‐27632 on proliferation of PASMCs treated for 24 h with either 10^−8^ mol/L ET‐1 or vehicle (*n* = 4–17 per group). Proliferation was measured by ELISA for BrdU incorporation. Bars show mean with standard error. *Indicates significant difference between groups (*P* < 0.05 by two‐way ANOVA with Tukey's test for multiple comparisons).

**Figure 4:**
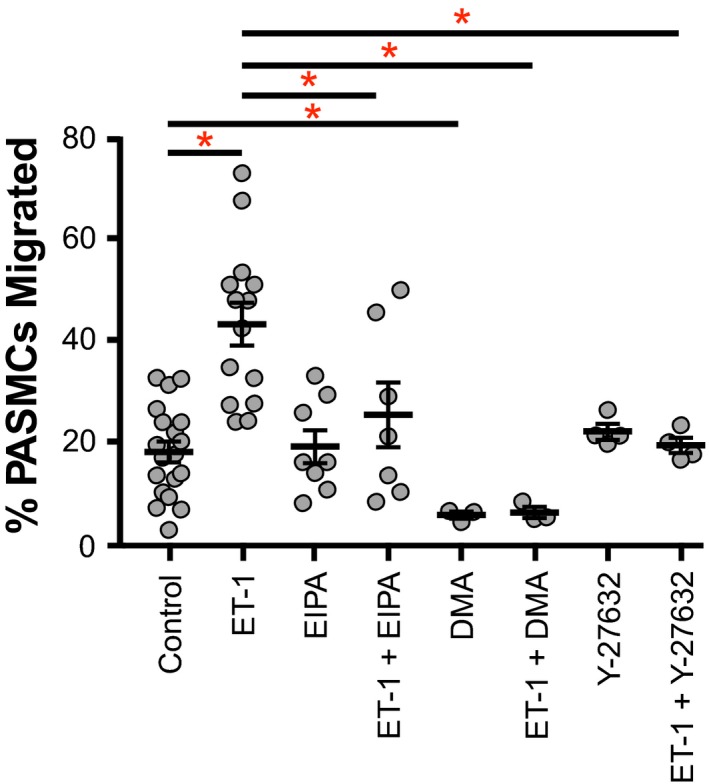
Effect of Na^+^/H^+^ exchanger (NHE) and Rho kinase inhibition on ET‐1‐induced migration of rat pulmonary arterial smooth muscle cells (PASMCs). Plot shows effect of 10 *μ*mol/L ethyl‐isopropyl amiloride (EIPA), 1 *μ*mol/L dimethyl amiloride (DMA), or 10 *μ*mol/L Y‐27632 on migration of PASMCs treated for 24 h with either 10^−8^ mol/L ET‐1 or vehicle (*n* = 3–19 per group). Migration was measured by Transwell assay. Bars show mean with standard error. *Indicates significant difference between groups (*P* < 0.05 by two‐way ANOVA with Tukey's test for multiple comparisons).

Since alterations in PASMC pH_i_ have been associated with changes in cell function (Quinn et al. 1996; Walker et al. 2016), we next wanted to determine whether ET‐1 affected PASMC proliferation and migration. Following ET‐1 treatment, proliferation in PASMCs was increased by 61 ± 13%, compared to vehicle‐treatment (Fig. 3). Similarly, ET‐1 increased PASMC migration (43.0 ± 4.1% migrated) compared to controls (17.9 ± 2.0% migrated) (Fig. 4). ET‐1‐induced cell alkalinization was therefore associated with increased proliferation and migration.

### NHE and PASMC function

Since ET‐1 increased both NHE activity and proliferation/migration in PASMCs, we next assessed whether the ET‐1 effect on PASMC function was dependent on NHE activation. The ET‐1‐induced increase in PASMC proliferation (to 161 ± 13% of control) was prevented by pharmacologic inhibition of NHE with EIPA (resulting in proliferation of 65 ± 6% of control) or with DMA (84 ± 30% of control) (Fig. 3). Of note, EIPA also significantly decreased proliferation of PASMCs treated with vehicle alone (58 ± 14% of control), whereas DMA did not have a significant effect on vehicle‐treated PASMCs. Similarly, the ET‐1‐induced increase in PASMC migration did not occur in the presence of EIPA (25.3 ± 6.4% migrated) or DMA (5.5 ± 0.7% migrated) (Fig. 4). Surprisingly, EIPA did not decrease migration in vehicle‐treated cells, whereas DMA did. We have previously shown that the EIPA/DMA vehicle, DMSO, does not significantly alter PASMC proliferation or migration (Walker et al. 2016). These data indicate that ET‐1‐induced increases in PASMC proliferation and migration require NHE.

### ET‐1 effect on NHE1 expression

**Figure 5:**
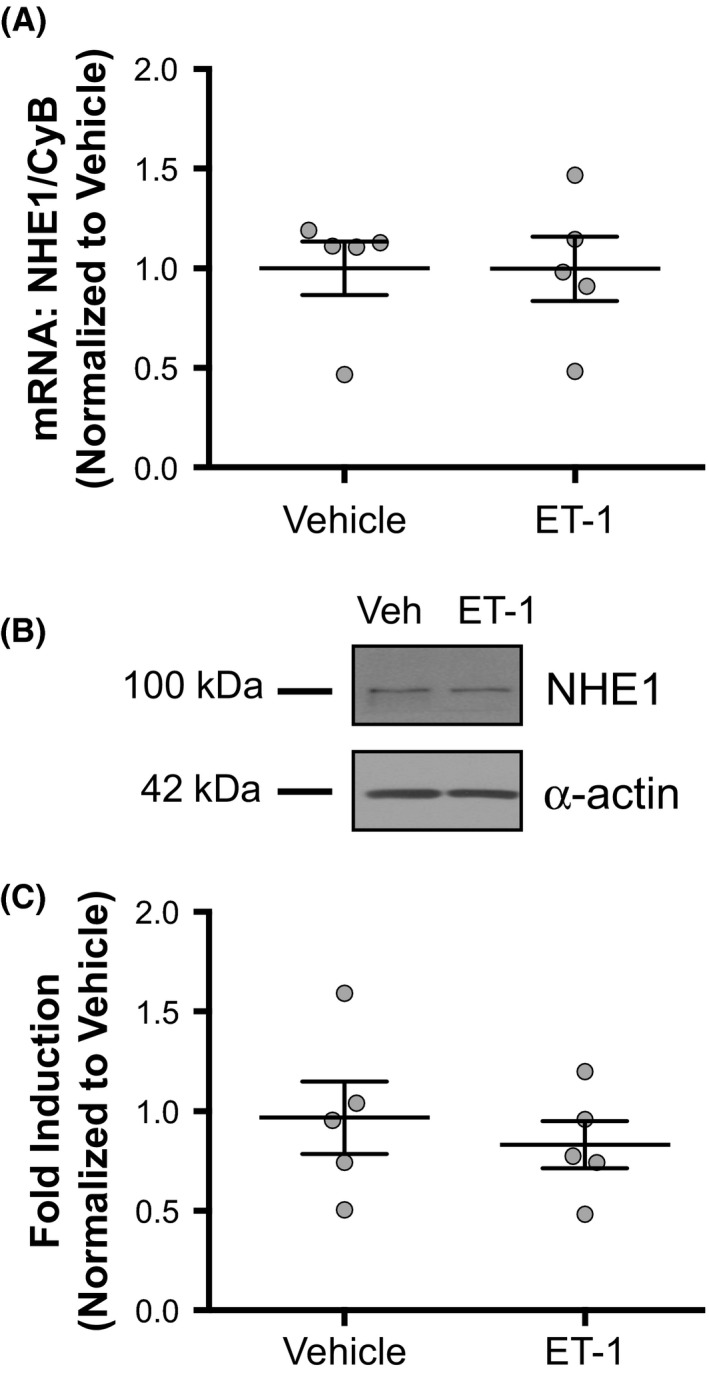
Effect of ET‐1 on Na^+^/H^+^ exchanger 1 (NHE1) expression in rat pulmonary arterial smooth muscle cells (PASMCs). (A) Plot shows quantitative RT‐PCR measurement of NHE1 mRNA expression relative to housekeeping gene cyclophilin B (CyB) in PASMCs treated for 24 h with 10^−8^ mol/L ET‐1 or vehicle (*n* = 5 for each), with the mean expression ratio of vehicle group set to 1. (B) Representative immunoblot shows NHE1 and *α*‐smooth muscle actin protein expression in vehicle (veh)‐ and ET‐1 treated PASMCs. (C) Plot shows quantification of immunoblot measurement of NHE1 protein expression normalized to *α*‐actin expression in PASMCs treated for 24 h with 10^−8^ mol/L ET‐1 or vehicle (*n* = 7 for each), with the mean expression ratio of vehicle group set to 1. Bars show mean with standard error.

Hypoxia‐induced increases in NHE1 expression have been found to be mediated by ET‐1 (Pisarcik et al. 2013). Given our data showing that ET‐1 increased NHE activity, we wanted to determine whether this was due to increased NHE1 expression. NHE1 mRNA expression was not affected by ET‐1 treatment (Fig. 5A). Similarly, immunoblot measurement of NHE1 protein expression showed no difference between ET‐1 and vehicle‐treated PASMCs (Fig. 5B and C). These results indicate that the effect of ET‐1 on NHE activity was not mediated by increased NHE1 expression.

### ROCK and PASMC function

Acute exposure to ET‐1 increases NHE activity via ROCK (Undem et al. 2012). Therefore, we assessed whether the effect of ET‐1 treatment on PASMC proliferation/migration was dependent on ROCK. Pharmacologic inhibition of ROCK with Y‐27632 prevented the ET‐1‐induced increase in PASMC proliferation (96 ± 11% of control) (Fig. 3). ROCK inhibition had no effect on vehicle‐treated cells. Analogously, ROCK inhibition blunted the ET‐1 effect on PASMC migration (19.3 ± 1.5% migrated) but had no effect on vehicle‐treated cells (Fig. 4). These data indicate that ET‐1 activation of PASMC proliferation and migration is dependent on ROCK.

## Discussion

The potent vasoconstrictor effect of ET‐1 has long been recognized. Potentially less appreciated is a smaller body of work indicating that ET‐1 can promote PASMC proliferation (Janakidevi et al. 1992; Zamora et al. 1996; Wort et al. 2001) and migration (Meoli and White 2010), processes important to vascular remodeling in PH. In this study, we demonstrated that prolonged exposure to ET‐1 increased resting pH_i_ and NHE activity in rat PASMCs. We also showed that ET‐1 enhanced PASMC proliferation and migration in vitro. Moreover, the increase in NHE activity was required for the ET‐1‐induced changes in PASMC function, which were also dependent on ROCK activation.

The results from this study show that prolonged ET‐1 treatment increased resting pH_i_ and NHE activity in rat PASMCs. Measurement of resting pH_i_ in this study corresponds well with previous reports of resting pH_i_ in rat PASMCs (Shimoda et al. 2006; Huetsch et al. 2016). Prior work in murine PASMCs showed a similar resting pH_i_ as well as similar increases in pH_i_ and NHE activity following acute ET‐1 exposure (Undem et al. 2012). Thus, the present results indicate that the effect of ET‐1 on cellular pH_i_ is not restricted to a single species and can be sustained in the setting of ongoing ET‐1 exposure. Of note, ET‐1 signaling effects may outlive the relatively short ET‐1 half‐life due to internalization of ET‐1‐bound receptors (Westcott et al. 1990); this is suggested by data showing that ET‐1‐induced vasoconstriction can persist long after removal of ET‐1 from the perfusing solution (Chatfield et al. 1991). Furthermore, our finding that ET‐1 increased resting pH_i_ in bicarbonate‐containing solutions (allowing membrane bicarbonate transporters, in addition to NHE, to participate in pH_i_ regulation) suggests that ET‐1 is able to induce changes in pH_i_ in physiologic solutions comparable to which PASMCs may be exposed in vivo.

Our current findings extend our prior work in that we now show that the effect of ET‐1 on PASMC pH_i_ has functional consequences in the form of increased proliferation and migration. These results complement a prior body of work indicating that increases in pH_i_ and NHE activity, in response to varied stimuli associated with the development of PH including growth factors, hypoxia, and SU5416‐hypoxia exposure (Quinn et al. 1996; Huetsch et al. 2016; Walker et al. 2016), are associated with enhanced PASMC proliferation and migration. Inhibition of NHE in vivo, either by genetic deletion (Yu et al. 2008; Walker et al. 2016) or pharmacologic inhibition (Quinn et al. 1998), has been shown to prevent the development of hypoxia‐induced vascular remodeling and PH, suggesting that regulation of cellular pH_i_ is not only relevant to PASMC function in vitro but is also a critical pathway through which vascular remodeling is mediated in vivo.

We have previously shown that long‐term exposure (48 h) of rat PASMCs to ET‐1 increased protein levels of the *α* subunit of hypoxia‐inducible factor 1 (HIF‐1) and increased mRNA expression of multiple HIF‐1 targets, including NHE1 (Pisarcik et al. 2013). The results in this study indicate that briefer exposure (24 h) to ET‐1 was not sufficient to induce NHE1 expression, but nonetheless resulted in both NHE activation and changes in PASMC function. We examined NHE1 expression in particular because we demonstrated that NHE1 is expressed in rat PASMCs while NHE2 and NHE3 are not (Shimoda et al. 2006). NHE3‐5 expression is absent in whole lung tissue preparations and NHE 6–10 are believed to have more restricted tissue and/or organellar‐specific localization (Huetsch and Shimoda 2015). Of note, the NHE inhibitors (EIPA and DMA) we used to assess the role of NHE activity in ET‐1‐induced changes in PASMC function inhibit the entire family of NHE isoforms (Masereel 2003) and at the doses used (10 *μ*mol/L EIPA and 1 *μ*mol/L DMA) have been previously shown to decrease global NHE activity in PASMCs (Rios et al. 2005; Undem et al. 2012). Thus, while the data collectively suggest that the effect of ET‐1 is likely mediated by NHE1, we cannot entirely rule out the possibility that ET‐1‐induced upregulation of NHE activity was achieved through increased expression of an alternate NHE family member.

Our results show a discrepant effect of EIPA and DMA upon basal PASMC function, such that EIPA significantly decreased proliferation and DMA decreased migration. While the explanation for this discrepancy is not entirely clear, it may be related to intragroup variability. For example, there is significant variability in control PASMC migration rates, and DMA or EIPA were each used to treat only a subset of these cells. In the subset of PASMCs in which the effect of DMA upon migration was tested (n = 3), both vehicle‐treated and DMA‐treated migration rates were low and were not significantly different (*P *=* *0.81 by *t*‐test).

Since ET‐1 increased NHE activity without increasing NHE1 levels, we examined other potential mechanisms that could mediate an increase in NHE activity and lead to the ET‐1‐induced changes in PASMC function we observed. Phosphorylation of the NHE cytoplasmic C‐terminal tail by a wide range of kinases, including ROCK (Tominaga et al. 1998; Wallert et al. 2015), is a well‐described mechanism of NHE activation (Huetsch and Shimoda 2015). Indeed, we previously showed that ET‐1 acutely increased NHE activity in PASMCs via ROCK activation (Undem et al. 2012), providing a mechanism whereby NHE activation occurs in the absence of altered NHE1 expression. As with NHE inhibition, we found that ROCK inhibition prevented ET‐1‐induced changes in PASMC function. 10 *μ*mol/L Y‐27632 was used to inhibit ROCK because this dose has both been shown to significantly decrease (>50%) ROCK activity in PASMCs (Luke et al. 2012) and because this dose has been shown to abrogate ET‐1‐induced increases in NHE activity while having no significant effect on NHE activity in unstimulated control PASMCs (Undem et al. 2012). Interestingly, inhibiting ROCK had no effect on migration or proliferation in control cells, indicating that either ROCK activation is low at baseline or, if active, does not contribute meaningfully to basal migration/proliferation rates. Additionally, we have shown that ROCK activity was necessary for hypoxia‐induced increases in PASMC proliferation and migration (Walker et al. 2016). We speculate that the effects of ROCK during hypoxia may result from increased ET‐1 production under these conditions (Whitman et al. 2008) and thus, could reflect hypoxia‐induced activation of the pathway described in this paper. However, whether ET‐1 mediates the effects of hypoxia on PASMC migration and proliferation remains to be determined.

While we show that the effects of ET‐1 on PASMC function were blocked when ROCK activation was inhibited, a question still remains as to whether ROCK‐dependent NHE activation in PASMCs is due to a direct ROCK‐NHE interaction and, if so, which residue(s) on NHE might be phosphorylated. Threonine 653 on NHE1 is a residue of interest, as phosphorylation of this site by ROCK in fibroblasts results in increased ion transport activity and migration (Wallert et al. 2015). Future studies will be needed to determine if this interaction also occurs in PASMCs and contributes to ET‐1‐induced activation of NHE.

While the results of this study use an in vitro model to describe the effects of ET‐1 on PASMC function, our results may have implications for PASMC function in vivo. ET‐1 signaling has been an area of intense interest in PH, as ET‐1 inhibitors have been shown to be efficacious in multiple preclinical models of PH (Eddahibi et al. 1995; Chen et al. 1997; Rafikova et al. 2013), and have become a mainstay of therapy for pulmonary arterial hypertension (Galie et al. 2013). Prior work has shown that ET‐1, via stimulation of the ET_A_ receptor, activates ROCK in PASMCs with resultant heightened vasoconstriction (Homma et al. 2007). Moreover, ROCK inhibitors have been used to effect acute pulmonary vasodilation in multiple models of PH (Nagaoka et al. 2004; Oka et al. 2007). Although most prior work has focused on the vasoconstrictor effects of ET‐1 and ROCK, ROCK inhibition has been effective in reducing pulmonary vascular remodeling in multiple preclinical models of PH (Mouchaers et al. 2010; Xu et al. 2010). The data in this study further support the concept that the ET‐1/ROCK signaling pathway contributes not only to vasoconstriction but also to changes in PASMC proliferation and migration, and elucidate a mechanism, involving NHE activity, by which prolonged ROCK inhibition can result in alterations in cell function that may impact the vascular remodeling process.

In summary, in this study we demonstrate that prolonged challenge with exogenous ET‐1 enhanced PASMC pH_i_ and NHE activity, and that ET‐1‐induced increases in PASMC migration and proliferation are mediated by NHE and ROCK. Given the known involvement of ET‐1 in the pathogenesis and progression of PH, these results may provide additional insight into the mechanism by which ET‐1 modulates vascular muscularization under both physiological and pathologic conditions.

## Data Sharing

Data included in this manuscript will be made available via Figshare.
